# Mycobacterium tuberculosis-Associated Serpiginous-Like Choroiditis Mimicking Central Serous Chorioretinopathy: A Case Report

**DOI:** 10.7759/cureus.107575

**Published:** 2026-04-23

**Authors:** Soraya A Horowitz, Nadyr Damasceno, Marcio Rodrigues, Adroaldo Alencar, Eduardo Damasceno

**Affiliations:** 1 Ophthalmology, Hospital Naval Marcilio Dias, Rio de Janeiro, BRA; 2 Ophthalmology, Universidade Federal do Rio de Janeiro, Rio de Janeiro, BRA; 3 Ophthalmology, Universidade Federal Fluminense, Niteroi, BRA

**Keywords:** central serous maculopathy like, serpiginous choroiditis, serpiginous-like choroiditis, sudden decrease in visual acuity, tuberculous retinitis

## Abstract

We report a case of a 40-year-old man with no comorbidities who presented with progressive vision loss in the right eye. Fundoscopy revealed peripapillary and posterior pole serpiginous-like lesions without overt vitritis. The central optical coherence tomography revealed subretinal fluid consistent with central serous chorioretinopathy, prompting treatment with spironolactone 100 mg daily for two months, which did not improve the condition. In the meantime, a positive tuberculin skin test indicated *Mycobacterium tuberculosis* infection, and a diagnosis of tuberculosis serpiginous-like choroiditis was assumed, leading to the initiation of specific antitubercular therapy with progressive improvement.

## Introduction

Serpiginous choroiditis is a rare, progressive, recurrent posterior uveitis that is typically bilateral but asymmetric, affects males and females equally, and usually presents between the third and sixth decades of life. It may be associated with flat subretinal fluid. In contrast, serpiginous-like choroiditis caused by *Mycobacterium tuberculosis* (MTB) is more often unilateral and shows a male predominance in adults. The diagnosis of tuberculosis (TB)-related disease depends on tests for MTB infection, including tuberculin skin test (TST), interferon-gamma release assays (IGRAs), and chest imaging; in certain instances, a presumptive diagnosis is made based on supportive tests within a fitting clinical context. It is crucial to rule out TB in patients with suspected serpiginous choroiditis, as treatment involves immunosuppressive agents, which are contraindicated in TB. These patients are treated with anti-TB drugs and systemic corticosteroids. Another key differential diagnosis, particularly relevant in this case, is central serous chorioretinopathy (CSCR). Notably, chronic CSCR may resemble serpiginous choroiditis, as it can present with widespread retinal pigment epithelial (RPE) damage, areas of atrophy, and shallow subretinal fluid. Furthermore, CSCR may be precipitated or exacerbated by corticosteroid use, potentially creating a diagnostic and therapeutic challenge, as inappropriate corticosteroid administration may worsen the condition and lead to poor visual outcomes [1-7].

## Case presentation

A 40-year-old man presented with a two-week history of progressive vision loss in his right eye (RE). Best-corrected visual acuity (BCVA), measured using a Snellen chart, was 20/100 in the RE and 20/20 in the left eye (LE). Slit-lamp biomicroscopy of the anterior chamber was unremarkable in both eyes. Intraocular pressure was within the normal limits in both eyes. 

**Figure 1:**
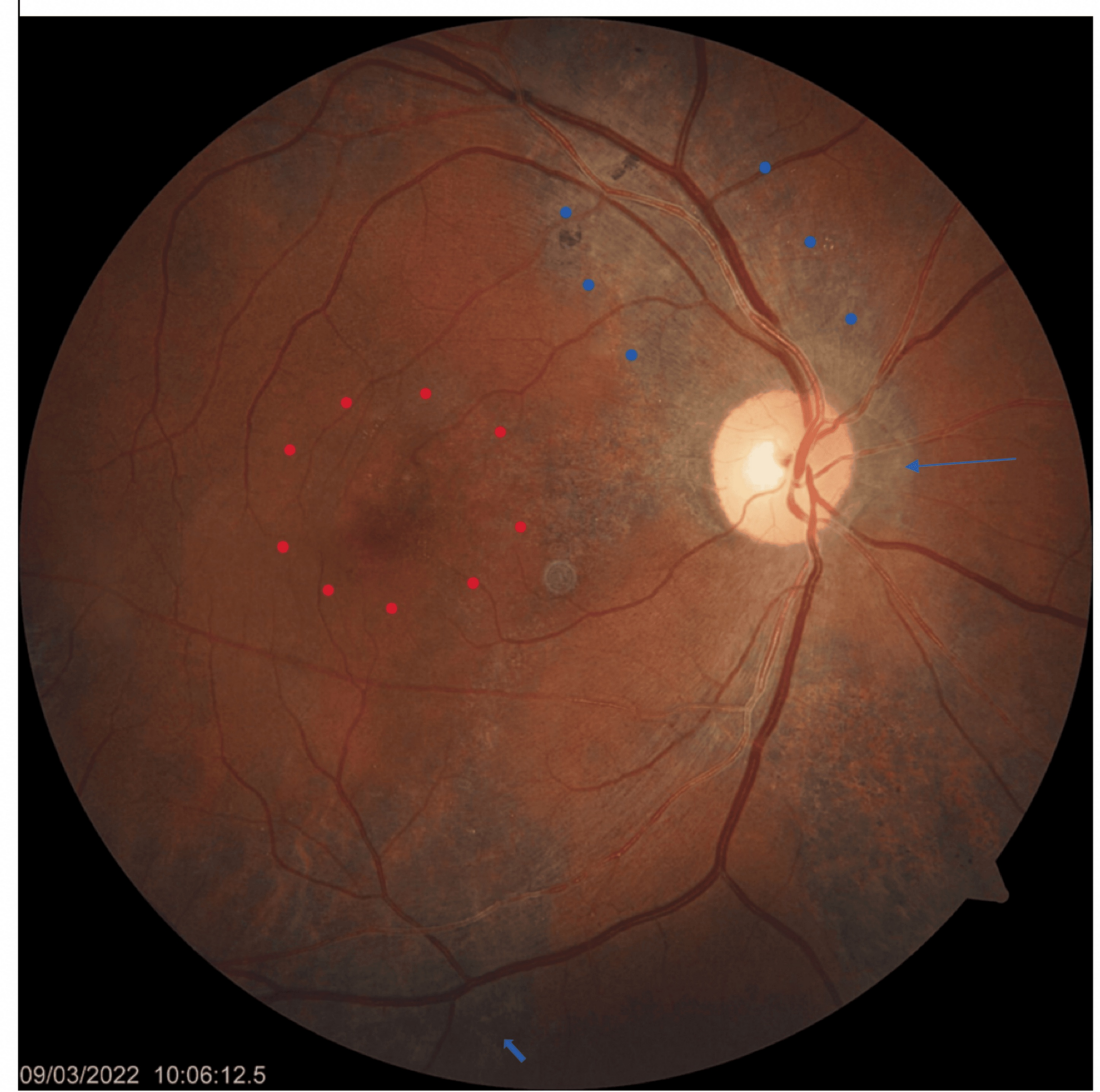
Color fundus photograph of the RE showing multiple gray-yellowish lesions at the macula, surrounded by areas of hyperpigmentation (red dots). Confluent peripapillary lesions (thin blue arrow) extend centrifugally toward the superior (blue dots) and inferior retina (thick blue arrow). RE: right eye.

Fundoscopy revealed multiple gray-yellowish lesions at the macula, surrounded by areas of hyperpigmentation. There were confluent peripapillary lesions extending centrifugally toward the superior and inferior retina, with coexistence of active gray-yellow lesions and inactive hyperpigmented scars, and no vitreous cells in the RE (Figure 1). The LE was within normal limits.

**Figure 2:**
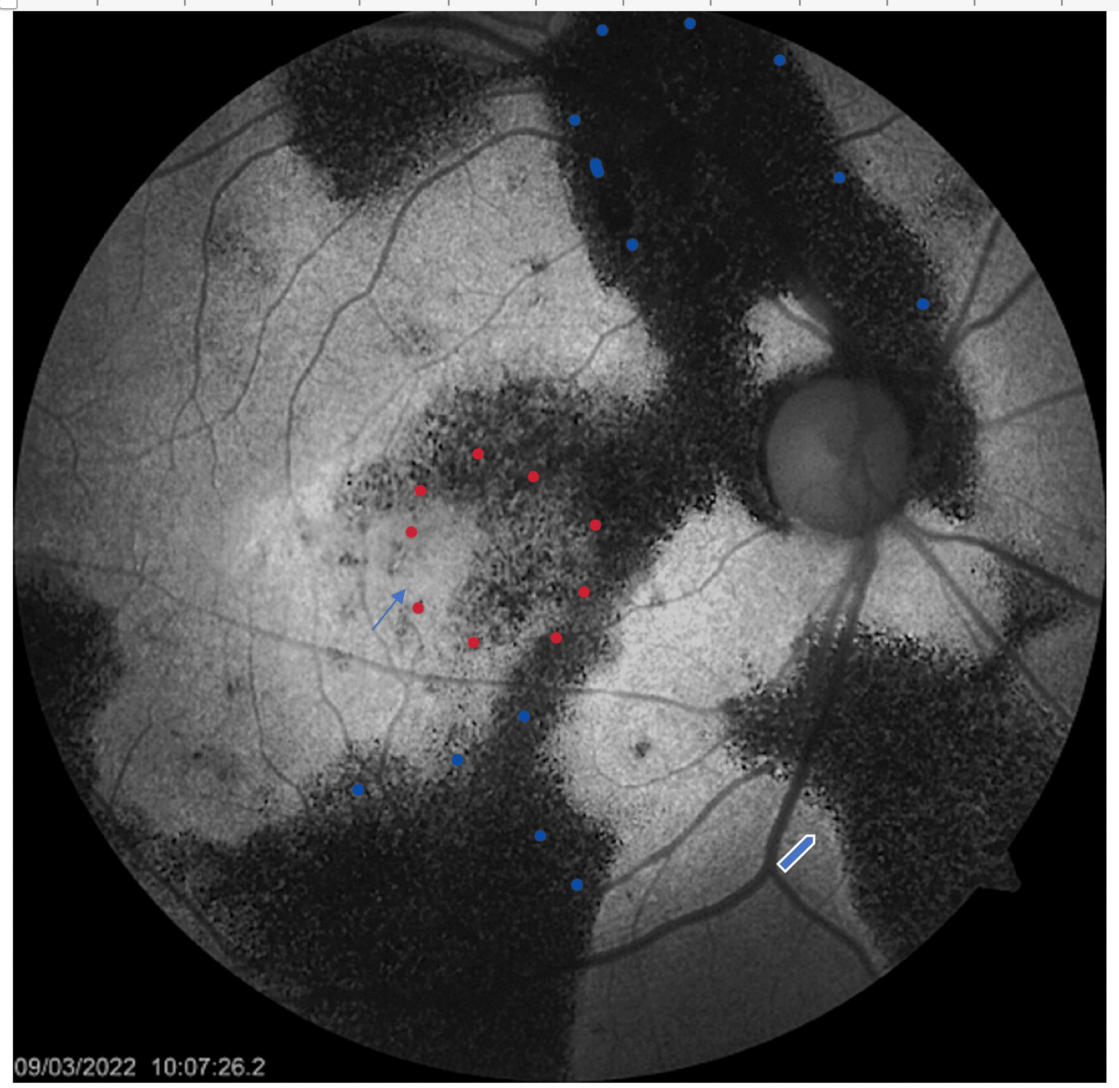
Fundus autofluorescence shows hypoautofluorescent peripapillary lesions extending to the superior and inferior retina (blue dots) and to the macula (red dots). These lesions have a thin hyperautofluorescent border in some areas (thick blue arrow). There is hyperautofluorescence in the temporal macula (thin blue arrow).

In the RE, the lesions were predominantly hypoautofluorescent with thin hyperfluorescent border in some areas. The temporal macula was hyperautofluorescent (Figure 2).

**Figure 3:**
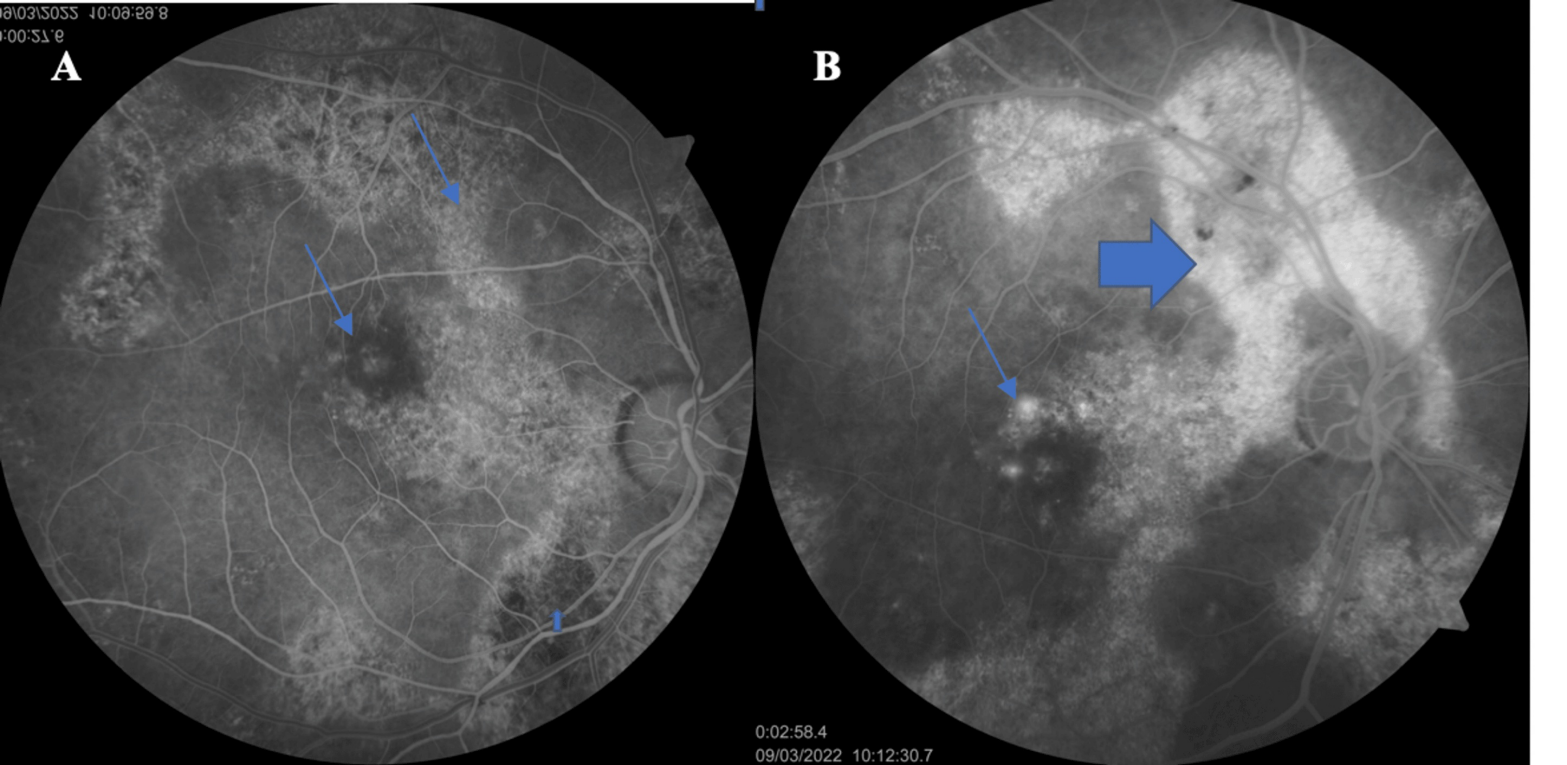
(A) An early-phase FA of the of RE shows window defect hyperfluorescence of the central macular and extensive lesions (thin blue arrows), and a hypofluorescent spot (thick blue arrow). (B) The late-phase FA of the RE shows leakage hyperfluorescence of the macula (thin blue arrow) and extensive staining hyperfluorescence of the superior retina (thick blue arrow).

Figure 3A shows the early phase of the fluorescein angiography (FA) in the RE. In the early phase, there is an extensive peripapillary window defect hyperfluorescent lesion in a serpiginous pattern extending to the superior and inferior retina and macula, due to RPE atrophy, with areas of hypofluorescence due to lesion activity. In the macula, a central window defect hyperfluorescent lesion is seen, surrounded by hypofluorescence due to lesion activity. In the late phase, there are hyperfluorescent areas due to staining in the superior retina (indicating inactivity) and three spots with leakage in the macula (Figure 3B). This represents typical serpiginous choroiditis with a mixed early-phase FA. Inactive lesions show window defect hyperfluorescence, while active lesions are hypofluorescent.

**Figure 4:**
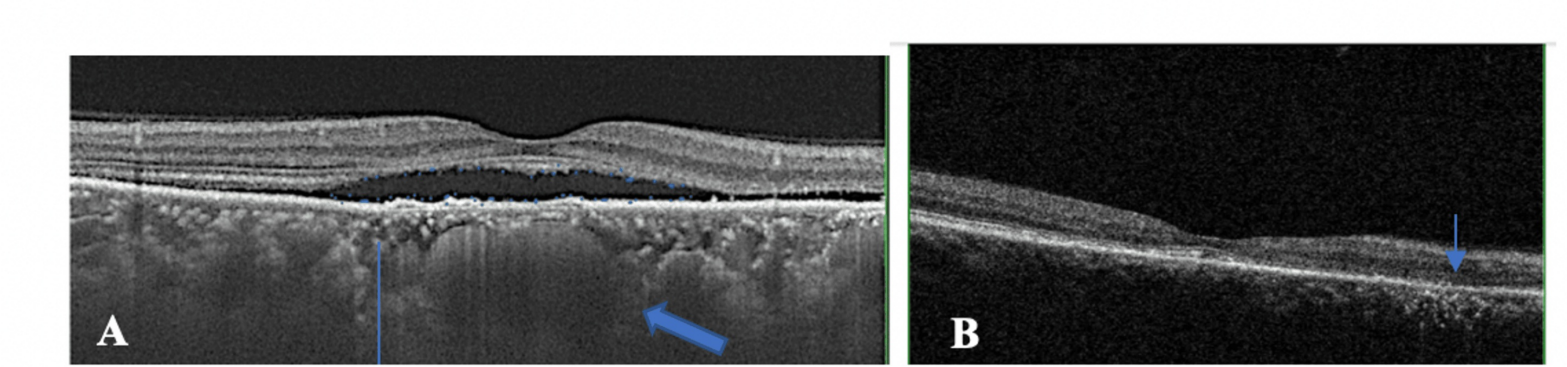
OCT of the RE during active disease showing subretinal fluid (blue dots surround a hyporeflective space), increased choroidal thickness (thin blue vertical line), and pachyvessel (thick blue arrow). (B) OCT of the RE post-treatment (inactive) showing an area of outer retinal rupture and RPE atrophy (blue arrow).

Optical coherence tomography (OCT) of the RE showed serous subretinal fluid, increased choroidal thickness, and pachyvessels. The RPE presented discrete hyperreflective dots at the level of the RPE, small RPE detachments, and central RPE thinning. There were irregularities in the ellipsoid zone (Figure 4A). The OCT alterations were compatible with chronic CSCR.

He was initially treated for CSCR with spironolactone 100 mg daily for two months, but showed no improvement. The peripapillary lesions extending centrifugally in a snake-like pattern suggested the coexistence of serpiginous choroiditis. Given the need for immunosuppressive agents, examinations were required to rule out infectious choroiditis. 

He underwent uveitis screening (VDRL, FTA-ABS, TST, and IgM/IgG for toxoplasmosis) and had a reactive TST (>15 mm) with normal chest imaging. He did not live in an endemic area for tuberculosis. The diagnosis of presumed tuberculosis serpiginous-like choroiditis was made based on the positive TST and lesion characteristics. Notably, increased choroidal thickness can occur in both chronic CSCR and TB serpiginous-like choroiditis, unlike typical serpiginous choroiditis. 

He received anti-TB therapy (isoniazid, rifampin, ethambutol, and pyrazinamide) for nine months and corticosteroid (60 mg/day, tapered gradually over three months). The lesions healed, and subretinal fluid resolved, leading to an area of rupture of the outer retina and RPE atrophy within two months. BCVA was 20/40 at the last visit (Figure 4B).

## Discussion

This case highlights the importance of distinguishing between tuberculosis-associated serpiginous-like choroiditis and chronic central serous chorioretinopathy (cCSCR), as their treatment strategies differ significantly, and misdiagnosis may lead to worse visual outcomes.

The main diagnostic challenge was differentiating serpiginous choroiditis from cCSCR, as both conditions may present with retinal pigment epithelium (RPE) alterations and subretinal fluid. However, key differences in fluorescein angiography (FA) patterns helped guide the diagnosis. In CSCR, FA typically demonstrates focal leakage points with progressive “inkblot” or “smokestack” expansion and pooling of dye in the subretinal space. In contrast, our case showed extensive peripapillary hyperfluorescent window defects in a serpiginous distribution, corresponding to RPE atrophy, associated with adjacent hypofluorescent areas and late staining hyperfluorescence in the superior retina, along with three leakage points in the macula. Additionally, in cCSCR, RPE damage is usually related to chronic subretinal fluid with a gravitational pattern of distribution, which was not the predominant feature in this case [8,9].

Serpiginous choroiditis is an important differential diagnosis, characterized by lesions that typically originate in the peripapillary region and extend centrifugally in a serpiginous (snake-like) pattern toward the macula and peripheral retina. It is a rare, chronic, progressive, usually bilateral but asymmetric inflammatory disease involving the choroid and RPE. Inflammatory signs such as vitritis or anterior chamber reaction are typically minimal or absent [2,10].

On optical coherence tomography (OCT), active serpiginous choroiditis usually demonstrates hyperreflectivity of the photoreceptor ellipsoid and myoid zones, disruption of the external limiting membrane and interdigitation zone, and RPE alterations. Healed lesions are characterized by outer retinal atrophy, RPE clumping, and choriocapillaris thinning. These findings were not observed in this case. Although the coexistence of both entities is possible, the initial working diagnosis was atypical cCSCR, and treatment with oral spironolactone was initiated. Subthreshold micropulse yellow laser (577 nm) is another therapeutic option for cCSCR [8,10].

Among infectious etiologies, syphilis remains the “great mimicker” and may present with similar geographic chorioretinal lesions. Other considerations include atypical toxoplasmosis and viral infections such as herpes. Additional differential diagnoses include inflammatory choroiditides such as ampiginous chorioretinopathy, which presents with multifocal plaque-like lesions in the posterior pole, and acute posterior multifocal placoid pigment epitheliopathy (APMPPE), which is typically self-limited and non-recurrent [10,11].

The most important differential diagnosis of serpiginous choroiditis is presumed tuberculosis-associated serpiginous-like choroiditis. This condition is usually supported by chest imaging and immunologic tests such as the tuberculin skin test (TST) and interferon-gamma release assays (IGRAs), as definitive microbiological confirmation is rarely achieved due to the paucibacillary nature of *Mycobacterium tuberculosis*. Clinically, tuberculosis-associated serpiginous-like choroiditis often presents with multifocal lesions that are noncontiguous with the optic disc, may involve the retinal periphery, tend to spare the macula in early stages, and are more frequently associated with vitritis. On OCT, increased choroidal thickness may help distinguish it from idiopathic serpiginous choroiditis [12].

Establishing the correct diagnosis is critical due to its therapeutic implications. Infectious choroiditides require targeted antimicrobial therapy, whereas noninfectious entities such as serpiginous and ampiginous choroiditis are treated with immunosuppressive agents. Importantly, corticosteroids, commonly used in inflammatory conditions, may worsen cCSCR. In contrast, tuberculosis-associated choroiditis requires anti-tubercular therapy combined with corticosteroids [13]. In this case, the lesions resolved and visual acuity improved following anti-tubercular treatment.

Tuberculosis-associated serpiginous-like choroiditis may be misdiagnosed as cCSCR, particularly in its diffuse retinal pigment epitheliopathy variant. Conversely, cCSCR may be mistaken for inflammatory or infectious choroiditis. Such diagnostic confusion may lead to inappropriate treatment and poorer visual outcomes [14].

The diagnosis of ocular tuberculosis remains challenging and is typically based on a combination of clinical findings and supportive investigations. Classification systems categorize cases as “confirmed,” “probable,” or “possible.” A “confirmed” diagnosis requires microbiological identification of *M. tuberculosis* in ocular fluids or tissues, which is rarely feasible. A “probable” diagnosis is based on immunologic evidence (TST or IGRA) along with compatible clinical findings, while a “possible” diagnosis relies on clinical presentation and epidemiologic context, such as residence in endemic areas [5,7,15-17]. In this case, the diagnosis was supported by a positive TST and a favorable response to anti-tubercular therapy [7,18].

## Conclusions

Accurate diagnosis of choroiditis is essential, as inappropriate treatment may have devastating consequences, particularly in tuberculosis-associated serpiginous-like choroiditis. OCT is a valuable noninvasive tool for differentiating classic serpiginous choroiditis from tuberculosis-associated serpiginous-like choroiditis, as increased choroidal thickness is typically absent in the former. This case also highlights the importance of multimodal imaging, as fluorescein angiography demonstrated features consistent with serpiginous choroiditis, helping distinguish it from CSCR.
